# Proapoptotic and Antiangiogenic Activities of *Arctium Lappa* L. on Breast Cancer Cell Lines

**DOI:** 10.1155/2020/7286053

**Published:** 2020-05-17

**Authors:** Mohamad Taleb Agha, Hussein M. Baharetha, Majed Ahmed Al-Mansoub, Yasser M. Tabana, Nur Hidayah Kaz Abdul Aziz, Mun Fei Yam, Amin Malik Shah Abdul Majid

**Affiliations:** ^1^EMAN Testing & Research Laboratory, Department of Pharmacology, School of Pharmaceutical Sciences, Universiti Sains Malaysia, Minden 11800, Pinang, Malaysia; ^2^Department of Pharmacy, College of Medicine and Health Sciences, Hadhramout University, Al Mukalla, Hadhramout, Yemen; ^3^Faculty of Pharmacy and Pharmaceutical Sciences, University of Alberta, Edmonton, Alberta, Canada; ^4^ACRF Department of Cancer Biology and Therapeutics, The John Curtin School of Medical Research, Australian National University, Canberra, Australia

## Abstract

In this study, the bioactivity-guided fractionation was conducted on the aerial parts of *Arctium lappa* L. and then the extracts were tested *in vitro* on breast cancer (MCF-7), colorectal cancer (HCT-116), and normal cells (EA.hy926). The n-hexane fraction (EHX) of the ethanolic extract showed strong activity against both MCF-7 and EA.hy926 cell lines (IC_50_ values: 14.08 ± 3.64 and 27.25 ± 3.45 *μ*g/mL, respectively). The proapoptotic activity of EHX was assessed using MCF-7. Morphological alterations were visualized using Hoechst staining and a transmission electron microscope. Cancer cell signal transduction pathways were investigated, and EHX significantly upregulated p53, TGF-*β*, and NF-*κ*B. Furthermore, EHX was found to disrupt the metastatic cascade of breast cancer cells by the inhibition of cell proliferation, migration, invasion, and colonization. The antiangiogenic activity of EHX fraction showed potent inhibition of rat aorta microvessels with IC_50_ value: 4.34 ± 1.64 *μ*g/mL. This result was supported by the downregulation of VEGF-A expression up to 54%. Over 20 compounds were identified in EHX using GC-MS, of which stigmasterol, *ß*-sitosterol, and 3-O-acetyllupeol are the major active compounds. Phytochemical analysis of EHX showed higher phenolic and flavonoid contents with a substantial antioxidant activity. In conclusion, this work demonstrated that *A. lappa* has valuable anticancer activity and antiangiogenic properties that might be useful in breast cancer therapy.

## 1. Introduction

*Arctium Lappa* L. (*A. lappa*) is an edible plant and it is indigenous to North Asia and Europe. It is popular as a vegetable in Asian countries and it was used in many traditional European dishes. In China, it is used as a medicinal plant [1, 2]. The anticancer characteristics of *A. lappa* have been validated for extracts and isolated principles through various studies. Recent studies have found that *A. lappa* possesses a potent antiproliferative effect against various kinds of cancer cells such as human multiple myeloma (MM) [3], human bone marrow (k562) [4], breast (MCF-7), liver (Huh-7), oropharyngeal (HTB-43), and bladder (ECV-304) [5]. The major active ingredients of *A. lappa* are lignans, sterols, terpenoids, and polyphenols. Several studies revealed that *ß*-sitosterol (sterols) inhibited tumor growth and stimulated apoptosis in prostate cancer; in addition, it reduced the growth and metastasis in breast cancer cells [6]. Terpenoids, such as lupeol, are reported as a potent antiangiogenic drug. The lupeol is able to suppress the neovessel formation in nontumor models, such as CAM and rat cornea model, and in a xenograft tumor model. It also downregulates angiogenic genes such as MMP-2 and -9, VEGF-A, flt-1, and HIF-1*α* which are associated with tumorigenic conditions [7, 8].

Breast cancer is the most common cancer among women. In 2019, an estimated 268,600 women were diagnosed with breast cancer in the United States. Approximately 12.8% of American women will be diagnosed with breast cancer over their lifetime [9, 10]. Breast cancer is classified into three major subtypes based on the expression of the molecular markers such as estrogen receptor (ER), progesterone receptor (PR), and human epidermal growth factor receptor 2 (HER2). Hormone receptor-positive is the most commonly diagnosed of all breast cancer (70% of patients). The triple-negative breast cancer is more aggressive than the other subtypes and more likely to recur [10, 11]. Apoptosis and angiogenesis events are firmly controlled by complex molecular signaling systems [12]. Bcl-2 gene is found to be overexpressed in 70% of breast cancer cells, and it is linked to p53 gene downregulation. p53 has been referred to as “Guardian of the Genome” due to cell cycle arrest and apoptosis induction effect [13–15]. A TGF-*β* signaling pathway is identified as a double-edged sword, which can function as a tumor suppressor and oncogenic pathway. The overexpression of TGF-*β* in breast cancer cells is able to suppress tumor development markedly [16]. NF-*κ*B is an essential contributor to angiogenesis and cell death suppression. Nevertheless, several studies reported that NF-*κ*B activation impedes tumorigenesis in *in vivo* tumor models [17]. VEGF-A is a pivotal factor for the angiogenesis cascade. It regulates the neovascularization of several pathological impairments and diseases such as breast cancer [18].

In this work, the aerial parts of *A. lappa* were subjected to bioactivity-guided fractionation and the extract from n-hexane was tested *in vitro* and *ex vivo* for its proapoptotic activity and antiangiogenic properties.

## 2. Materials and Methods

### 2.1. Chemicals and Materials

The solvents used acetone, ethanol, ethyl acetate, petroleum ether, n-butanol, and n-hexane were produced by Riedel-de Haёn, Germany. The chemicals used in the cell culture study, that is, Dulbecco's modified Eagle medium, heat-inactivated fetal bovine serum, penicillin/streptomycin solution, Roswell park memorial institute medium, phosphate-buffered saline, trypsin, tetrazolium salt (3-(4,5-dimethylthiazol-2-yl)-2,5diphenyl tetrazolium bromide), and dimethyl sulfoxide, were produced by Sigma-Aldrich, Germany. Human breast cancer cells (MCF-7), colorectal cancer cells (HCT-116), and endothelial cells (EA.hy926) were purchased from American Type Culture Collection (ATCC, Manassas, VA, USA). The reagents for the *ex vivo* culture study (amphotericin B, aprotinin, fibrinogen, Earle's salt (M199), L-glutamine, gentamycin, thrombin, matrigel, and suramin) and Hoechst assay (Hoechst 33342 stain and para-formaldehyde) were purchased from Sigma-Aldrich, USA.

### 2.2. Plant Collection and Authentication

The aerial part of *A. lappa* was collected from the garden of medicinal plants of Damascus University, Damascus, Syria, in March 2015. A voucher specimen was authenticated by a botanist Mr. Shunmugam (code: 11328) and deposited at the Herbarium unit of the School of Biology, Universiti Sains Malaysia.

### 2.3. Extraction and Fractionation

The bioassay-guided crude extract of *A. lappa* was prepared by maceration method at 45°C for 48 h. Sequentially, three different solvents with different polarities were used starting with nonpolar solvent petroleum ether (PT), followed by the ethanol (ETH), and water (WT) [19]. The three extracts were then tested for MTT assay and the ETH extract showed the highest activity. The bioassay-guided fractionation was carried out by liquid-liquid separation and four solvents were used, that is, n-hexane (EHX), ethyl acetate (EEA), n-butanol (EB), and water (EW), respectively [20].

### 2.4. Cell Culture and Cell Lines

Human cell lines, HCT 116 (colorectal carcinoma), MCF-7 (hormone-sensitive breast cancer), and EA.hy926 (human endothelial cell), were obtained from ATCC, USA. Cells were cultured at a humidified atmosphere (37°C) with CO_2_ (5%) in a growth medium supplemented with 10% FBS and penicillin/streptomycin (1%). MCF-7 and EA.hy926 cells were cultured in DMEM (Gibco/Life technology, UK), while HCT-116 cells were cultured in RPMI-1640 (Sigma-Aldrich, USA).

### 2.5. Cell Viability

MTT assay was performed to assess the antiproliferative effect of *A. lappa* extracts and fractions on HCT-116, MCF-7, and EA.hy926 cells. The assay plates were read using a microtiter plate reader (Thermolab Systems, Finland) at 570 nm. Absolute ethanol was used as a negative control. Tamoxifen and betulinic acid were used as a positive control for MCF-7 and EA.hy926, respectively [21, 22].

### 2.6. Hoechst Stain Test

Nuclear chromatin condensation is a critical feature characterizing apoptosis. MCF-7 at a concentration of 1 × 10^5^ cells/mL per well was added to 24-well plate, and it was incubated for 24 h. The old medium was replaced and EHX was added at a concentration of 7.5, 15, and 30 *μ*g/mL or absolute ethanol for the control sample. The plate was reincubated for another 24 h. The medium was discarded, cells were rinsed by PBS, and para-formaldehyde 4% (w/v) was added for cell fixation. After 20 min, Hoechst 33258 stain (10 *μ*g/mL; Sigma) was added to each well (300 *μ*L/well), and the plate was incubated for a further 20 min. Microimages were snapped under 20x magnification using a fluorescence microscope (EVOS fl, USA) [23].

### 2.7. Morphological Changes in MCF-7 by Using a Transmission Electron Microscope (TEM)

MCF-7 cells were treated for 24 h with EHX or absolute ethanol. Cells were fixed with 0.1 M McDowell–Trump and stained with osmium tetroxide (1%). The cells were then solidified in agar (2%), cut into small slides, and dehydrated in ethanol followed by acetone. The slides were embedded in resin and infiltrated for five days in Suprr's mixture at 60°C and changed repeatedly every day. Subsequently, the strips were molded in resin molds and sliced into 0.1 *μ*m thickness. The ultrathin sections were stained with toluidine blue (Sigma-Aldrich, USA) and collected in copper grids. Consequently, it was doubly stained with uranyl acetate and lead citrate (Sigma-Aldrich, USA) [24]. Finally, the cells were photographed using TEM (EFTEM Libra 120, Carl-Zeiss, Germany) at 1250x magnification.

### 2.8. Cell Signal Pathways

MCF-7 cells were harvested at 50%–80% confluency. Cells were seeded (75 *μ*L/well) in a 96-well plate kit (Promega, USA) and incubated for 12 h. The old medium was aspirated and replaced with DMEM (75 *μ*l) containing EHX (10 *μ*g/mL) or the vehicle. The plate was incubated for 24 h. Then, each well received Dual-Glo® reagent (75 *μ*L), and the plate was incubated at room temperature for 1 h. Firefly luciferase reporter was measured by using a luminescence microplate reader (HIDEX, Finland). Dual-Glo® Stop and Glo® reagent was added to each well (75 *μ*L), and the plate was incubated at room temperature for 1 h. Renilla luciferase reporter was measured by using a luminescence microplate reader (HIDEX, Finland). The fold change in each pathway activity was measured according to the following formula:

Fold change in pathway activity = treated well ratio (Firefly/Renilla)/untreated well ratio (Firefly/Renilla).

### 2.9. Colony Formation Assay on MCF-7 Cell Line

The aim of this assay was to test the capability of extract to inhibit the cancer cell colonization [25, 26]. MCF-7 was seeded (2 mL/well) in a 6-well plate at a concentration of 500 cells/mL and incubated for 12 h. Old DMEM was replaced, and EHX was added at different concentrations (0.87–7 *μ*g/mL); tamoxifen was used as a positive control (10 *μ*g/mL), and absolute ethanol was used for negative control. After 48 h, the treatment was replenished by fresh DMEM every 3-4 days until the formation of large colonies (50 cells). The colonies were fixed and stained with crystal violet (0.2%) (Sigma-Aldrich, USA). The percentage of plating efficiency (PE%) and the percentage of surviving fraction were calculated.

### 2.10. Cell Invasion Assay of MCF-7 Cells

The invasion assay was performed to evaluate the ability to suppress transmigration of cancer cells in the metastasis stage. Matrigel was pipetted (1 : 1 DMEM) in 96-well plate and incubated for 45 min. Then, MCF-7 (150 *μ*L/well) was seeded at a concentration of 5000 cells/well. The plate was treated by EHX (7.5 and 15 *μ*g/mL) or absolute ethanol. After 24 h, images were captured for each well using a florescent microscope at 4x magnification.

### 2.11. Cell Migration Assay on MCF-7 Cells

The assay was conducted to evaluate the ability to stop cells from migration [27]. A confluent monolayer of MCF-7 was scratched in a 6-well plate. The plate was then treated by EHX at different concentrations (15 and 30 *μ*g/mL) or absolute ethanol. Subsequently, images were captured at 3 time points (0, 6, and 12 h). Finally, the pictures were analyzed using ImageJ® software at various points to measure the distance between the cells of both sides of the rip.

### 2.12. Experimental Animals

Sprague-Dawley male rats were provided by The Animal Research and Service Centre (ARASC) at Universiti Sains Malaysia. The animals were eight to twelve weeks old (200–230 g) and kept in transit in a quiet and clean room with suitable temperature, lighting system, and ventilation for few days before being sacrificed. The study protocol was approved by the Animal Ethics Committee, USM (approval reference number: 2016/(102) (782)).

### 2.13. *Ex Vivo* Rat Aortic Ring Assay

The three-dimensional (3D) assay was performed to estimate the antiangiogenic efficacy of compounds regarding the outgrowth sprouted of microvessels from the rat aortic ring model [28, 29]. Thoracic aortas were carefully excised and sectioned into rings of 1 mm thickness in the M199 medium (Gibco/Life technology, UK). Fresh rings were explanted directly in a 48-well plate and the lower layer was added (300 *μ*L/well). Each well received 10 *μ*L of thrombin (50 U/mL in normal saline) (Sigma-Aldrich, USA). The plate was incubated for 60 min, and then, the upper layer with treatment was added (300 *μ*L/well). The plate was incubated for four days. Later on, the upper layer was pipetted out carefully and replaced with a freshly prepared upper layer with similar treatments and the plate was incubated for another 24 h. Finally, the rings were photographed with 4x magnification using a microscope EVOS fl (digital inverted microscope, USA). The lower layer was composed of M199, fibrinogen 3mg/mL, aprotinin 5 *μ*g/mL, and L-glutamine 1% (v/v). The upper layer was composed of M199, FBS 20% (v/v), L-glutamine 1% (v/v), amphotericin B 1% (v/v), *ε*-aminocaproic acid 0.1% (w/v), and gentamycin 0.6% (v/v). The treatment was prepared in various concentrations (3–100 *μ*g/mL) of *A. lappa* for the calculation of IC_50_. Suramin (100 *μ*L/mL) and absolute ethanol were used as a positive control and a negative control, respectively.

### 2.14. VEGF

The treated MCF-7 cells were harvested and lysed by using Wizard® SV lysis buffer (Promega, Madison, USA). The assay was run by using the ELISA kit of human VEGF-165 according to the manufacturers' protocol (Raybio, USA).

### 2.15. Gas Chromatography-Mass Spectrometry (GC-MS)

ETH and EHX were carried out on a gas chromatograph HP 6890N (G1530N) (HP, China) attached with HP 5973 (G2579A) quadrupole mass spectrometer, at 70 eV. The stationary phase was packaged in a nonpolar capillary column HP-5MS 19091S-433. The initial temperature started at 70°C, for 2 min. Then, it was raised to 285°C at a rate of 20°C/min. The helium flow rate was 1.2 mL/min. The scan time and mass range were 1 s and 35–650° (*m*/*z*), respectively. Then, samples were injected (1 *μ*L) at the source temperature of 230°C while the temperature of the quadrupole column was 150°C. Then, mass spectra and mass/charge ratio (*m*/*z*) of molecular ions were compared to the referenced data of the NIST02 library [30].

### 2.16. Measurement of Total Phenol Content

The total phenol content of ETH and EHX was evaluated using Folin-Ciocalteu reagent as performed previously [31, 32]. The samples were prepared (1 mg/mL) of 100 *μ*L and mixed with 750 *μ*L of diluted Folin-Ciocalteu reagent in distilled water (1 : 10) and then incubated in the dark for 5 min at room temperature. Sodium bicarbonate solution (750 *μ*L) was added at a concentration of 60 g/L. Then, it was kept for 30 min in a dark environment at 30°C, and the absorbance was measured at 725 nm using a UV spectrophotometer (Perkin Lambda 45). The gallic acid was used as a standard.

### 2.17. Determination of Total Flavonoid Content

Total flavonoid content was evaluated using aluminum chloride method and quercetin was used as standard [33]. The samples (500 *μ*L in tubes) were prepared in a concentration of 1 mg/mL; then, we added 0.1 mL of 10% aluminum chloride solution, 1.5 mL of methanol, 0.1 mL of 1 M potassium acetate solution, and 2.8 mL of distilled water. After incubation for 30 min at room temperature, the absorbance reading was taken at 415 nm using a spectrophotometer (Perkin Lambda 45).

### 2.18. DPPH Free Radical Scavenging Assay

The samples were prepared (6–200 *μ*g/mL) and added to 100 *μ*L DPPH (1,1diphenyl-2-picrylhydrazyl) with a final concentration of 100 *μ*M DPPH. Absolute methanol was used as a negative control. All samples were incubated at 37°C, and then, the absorbance was measured by a microplate reader at 512 nm (Thermolab Systems, Finland). Ascorbic acid (Vit C) was used as a standard [34].

## 3. Results

### 3.1. Viability Assay

MTT assay was performed on two cancer cell lines and one normal cell line, i.e., human colorectal carcinoma cell line (HCT-116), human hormone-sensitive breast cancer cell line (MCF-7), and human hybrid endothelial cell line (EA.hy926). The screening test of *A. lappa* extracts (PT, ETH, and WT) showed that ETH was more active on MCF-7 compared to PT and WT extracts. The percentage of cell viability was 22.95 ± 1.01 (*p*< 0.05) at a concentration of 100 *μ*g/mL. Moreover, ETH inhibited the proliferation of EA.hy926 cells more than other extracts with a 35.53 ± 0.64% cell viability. Following fractionation, the screening test was performed on MCF-7 and EA.hy926 cell lines for all ETH fractions (EHX, EEA, EB, and EW) at a concentration 100 *μ*g/mL. The results show that EHX was significantly the most cytotoxic compared to other extracts and fractions. The percentage of cell viability on MCF-7 and EA.hy926 was 16.1 ± 1.81 and 22.97 ± 1.37 (*p* < 0.05), respectively (Table 1). The IC_50_ values are 14.08 ± 3.64 and 27.25 ± 3.45 *μ*g/mL for MCF-7 and EA.hy926, respectively. The positive control for MCF-7 was tamoxifen (IC_50_ 11.04 ± 1.31 *μ*g/mL), and that for EA.hy926 was betulinic acid (IC_50_ 4.27 ± 0.38 *μ*g/mL) (Table 2).

### 3.2. Proapoptotic Activity of EHX on MCF-7

#### 3.2.1. Morphological Changes of MCF-7 by the Hoechst Stain Test

**Figure 1:**
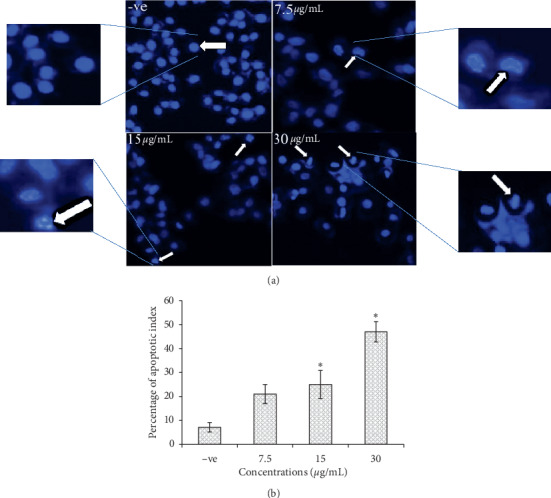
(a) MCF-7 cells in Hoechst stain demonstrate the normal morphology in the negative control (−ve) and the morphological changes in treated cells at different concentrations of EHX: kidney shape, apoptotic body, and cell shrinkage. Magnification is 20x. (b) Percentage of the apoptotic index at concentrations of 7.5, 15, and 30 *μ*g/mL. Values are mean ± SD. ^*∗*^*p* < 0.05.

This assay was conducted by using Hoechst 33258 dye which penetrates into cells' nuclei to stain the DNA [35]. The microimages illustrated manifestly cell shrinkage, kidney shape, and apoptotic body of the treated cells (Figure 1(a)). The apoptotic index was calculated for different concentrations (7.5, 15, and 30 *μ*g/mL), and the results indicated a dose-dependent relation with apoptotic values. The apoptotic index values were significantly (*p* < 0.05) different as 25.13 ± 5.87 and 47.97 ± 4.23% at concentrations of 15 and 30 *μ*g/mL, respectively, while the lower concentration (7.5 *μ*g/mL) showed no significant difference (21.58 ± 4%).

#### 3.2.2. Ultrastructural Changes of MCF-7 by TEM

**Figure 2:**
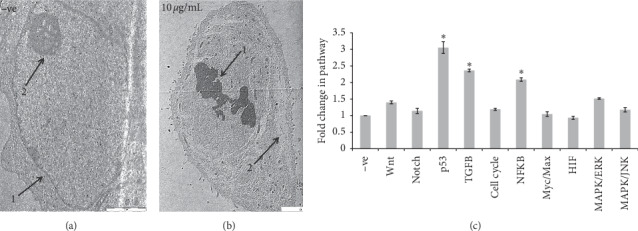
(a) TEM ultrastructural micrograph of untreated MCF-7 cells. 1. Nucleus. 2. Nucleolus. Magnification is 1600x. (b) Ultrastructural micrograph of MCF-7 cell after treatment by EHX (10 *μ*g/mL). 1: chromatin condensation; 2: vacuoles. Magnification is 1250x. (c) The effect of EHX (10 *μ*g/mL) on 10 signaling pathway activities. Values are mean ± SD. ^*∗*^*p* < 0.05.

TEM was performed to observe the ultrastructures of MCF-7. Cells were treated with EHX (10 *μ*g/mL) for 24 h, and microimages showed several signs of apoptosis induction compared to the control sample (Figures 2(a) and 2(b)). The normal cell morphology in the control sample was indicated as dense cellular content, diffused chromatin, and fewer vacuoles. On the other hand, the treated cell developed apoptotic changes such as condensed chromatin, floating nucleus content in the cytoplasm, apoptotic bodies, vacuoles with various sizes, and no nucleolus. This result suggests apoptotic cell death of MCF-7 cells upon EHX treatment [36].

#### 3.2.3. Cell Signal Pathways

A dual-luciferase reporter system was used to evaluate 10 signal pathways involved in apoptosis and angiogenesis process (Figure 2(c)). The data indicated that EHX upregulated 3 pathways significantly (*p* < 0.05). p53 (3.05-fold) is an important proapoptotic marker, TGF-*β* (2.36-fold) has tumor suppressor properties, and NFkB (2.08-fold) is an essential contributor in proliferation, metastasis, and angiogenesis. However, no significant change was observed in other pathways such as Wnt (1.4-fold), Notch (1.14-fold), cell cycle (1.19-fold), Myc/Max (1.04-fold), HIF (0.93-fold), MAPK/ERK (1.51-fold), and MAPK/JNK (1.17-fold).

#### 3.2.4. Colony Formation

**Figure 3:**
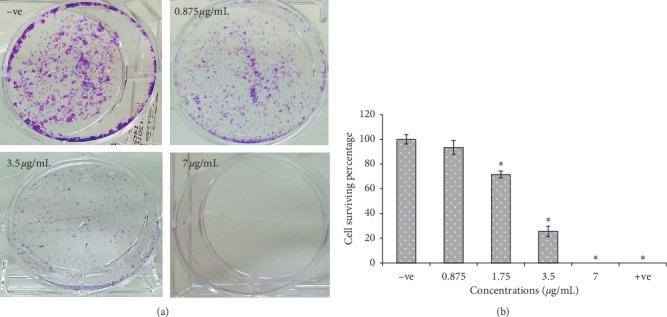
(a) MCF-7 cells in a colony formation assay. Cells were treated by ethanol (−ve) or EHX at different concentrations. (b) Percentage of cell surviving in colony formation assay. Tamoxifen (10 *μ*g/mL) was used as a positive control (+ve). Values are mean ± SD. ^*∗*^*p* < 0.05.

The colony formation assay was performed in a 6-well plate using MCF-7 cells at a concentration of 500 cell/mL (Figure 3(a)). The efficacy was presented as cell surviving percentage and the results were inversely proportional to EHX concentrations (0.875, 1.75, 3.5, and 7 *μ*g/mL). EHX showed zero percent colonization at 7 *μ*g/mL. The cell survival percentage was 71.52 ± 2.8 and 25.49 ± 4.21% for the concentrations of 1.75 and 3.5 *μ*g/mL, respectively (*p* < 0.05). However, there was no significant difference in cell survival percentage (93.37 ± 5.61%) for the concentration of 0.875 *μ*g/mL, compared to the negative control (Figure 3(b)). Tamoxifen (10 *μ*g/mL) was used as a positive control, and it inhibited cell colonization by 100%. Plating efficiency was 15.1 ± 0.56%.

#### 3.2.5. Cell Invasion

**Figure 4:**
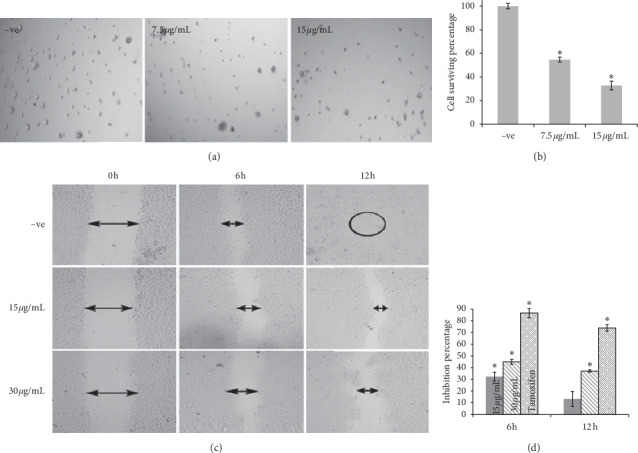
(a) Invasion assay of MCF-7 cells in matrigel basement. Cells were treated by ethanol (−ve) or EHX at different concentrations. Magnification is 4x. (b) Percentage of cell surviving in invasion assay. Values are mean ± SD. ^*∗*^*p* < 0.05. (c) Cell migration assay of MCF-7. Cells were treated by ethanol for negative control (−ve) or EHX at different concentrations. Wound healing was observed at time points 0, 6, and 12 hours. ImageJ software was used to measure the wound size. Magnification is 4x. (d) Percentage of inhibition of MCF-7 cells' migration after 6 and 12 hours of treatment. Tamoxifen (10 *μ*g/mL) was used as a positive control. Values are mean ± SD. ^*∗*^*p* < 0.05.

MCF-7 cell invasion was evaluated using a matrigel basement. The data was presented as a cell survival percentage at EHX concentrations (7.5 and 15 *μ*g/mL). Microimages exhibited a significant decrease in cell invasion in a dose-dependent manner (Figure 4(a)). Cell survival percentage was 45.35 ± 2.04 and 67.18 ± 3.75%, respectively (*p* < 0.05), compared to the control sample (Figure 4(b)).

#### 3.2.6. Cell Migration

The migration ability of MCF-7 was tested at two time points (6 h and 12 h) after EHX treatment at 15 and 30 *μ*g/mL. Wounds were created equally at zero hours for both concentrations (Figure 4(c)). After 6 h of treatment, cell migratory was inhibited significantly (*p* < 0.05) with values of 32.36 ± 3.7 and 45.02 ± 2.1%, respectively (Figure 4(d)), whereas the wound size became narrower compared to the untreated cells. After 12 h of treatment, no significant effect was indicated with 15 *μ*g/mL concentration, whereas higher concentration (30 *μ*g/mL) showed a significant inhibitory effect with a value of 37.14 ± 0.96% (*p* < 0.05). Tamoxifen was used as a positive control at a concentration of 10 *μ*g/mL. The untreated cells displayed more rapid wound closure at all time points, and the wound was completely closed after 12 h compared to the treated cells. The results suggested that EHX is able to inhibit *in vitro* cell migration of MCF-7 cells.

### 3.3. Antiangiogenic Activity

#### 3.3.1. Rat Aorta Ring Assay

**Figure 5:**
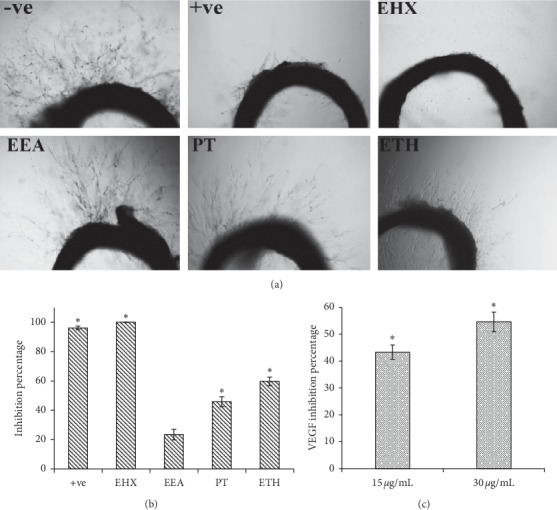
(a) Rat aortic ring assay. Rings were treated by ethanol (−ve), *A. lappa* extracts (100 *μ*g/mL), or Suramin (100 *μ*g/mL) (+ve). Microvessel outgrowth was measured by ImageJ software. Magnification is 4x. (b) Percentage of inhibition of microvessel outgrowth after treatment. Values are mean ± SD. ^*∗*^*p* < 0.05. (c) Percentage of inhibition of VEGF-A after EHX treatment (15 and 30 *μ*g/mL). Values are mean ± SD. ^*∗*^*p* < 0.05.

This assay was performed to evaluate the antiangiogenic activity of *A. lappa* extracts and fractions (Figures 5(a) and 5(b)). The data was presented as a percentage of inhibition of microvessel outgrowth from rat aortic rings. The extracts (PT, ETH, and WT) were initially tested at a single concentration of 100 *μ*g/mL to determine the most active one (Table 3). The ethanol extract (ETH) showed the most potent effect (58.75 ± 2.84%) in comparison with other extracts (*p* < 0.05). Following fractionation, all ETH fractions (EHX, EEA, EB, and EW) were screened at the same concentration (100 *μ*g/mL). Interestingly, EHX completely inhibited the microvessels sprouting activity (100%), and it was significantly (*p* < 0.05) the most active compared to the other fractions. The IC_50_ value of EHX was 4.34 ± 1.64 *μ*g/mL, and it was almost similar to the positive control (Suramin) with an IC_50_ value of 4.62 ± 1.17 *μ*g/mL.

#### 3.3.2. VEGF Expression by MCF-7

The EHX effect on rat aortic microvessels was supported by molecular findings specific to the angiogenesis process. The VEGF-165 protein expression was evaluated in MCF-7 cells using two concentrations of 15 and 30 *μ*g/mL. The results showed a significant (*p* < 0.05) inhibition in VEGF expression after 24 h (Figure 5(c)). The inhibition percentage of VEGF protein was 43.35 ± 2.65 and 54.61 ± 3.59%, respectively.

### 3.4. Phytochemical Study

#### 3.4.1. GC-MS

**Figure 6:**
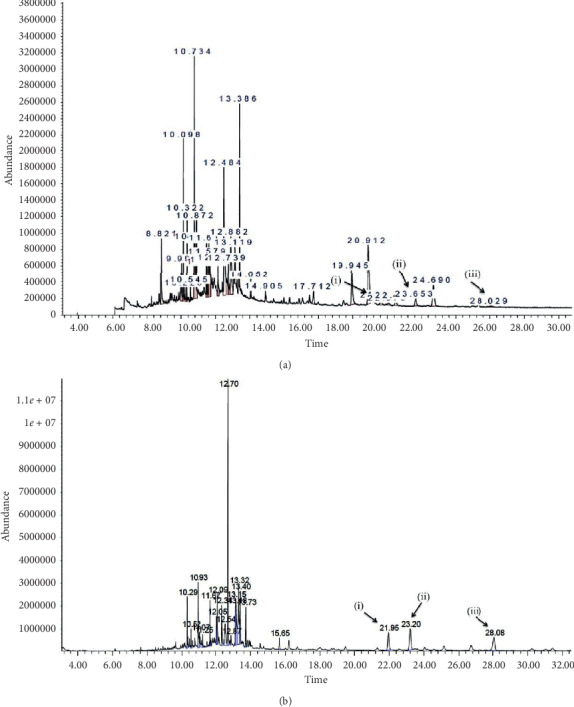
(a) and (b) Mass spectrogram of ETH extract and EHX fraction, respectively. (i) Stigmasterol. (ii) *ß*-Sitosterol. (iii) 3-O-Acetyllupeol.

GC-MS data of ETH and EHX was analyzed using MSD-ChemStation software (Agilent Technologies, USA). All compounds were identified by comparing to the reference of the NIST02 library (Figures 6(a) and 6(b)). Tables 4–6 elucidate (GC-MS) resulting chromatograms and mass spectra of probable constituents including major peaks of 22 identified compounds of ETH and 20 compounds of the EHX; they include the retention time (RT), percentages of area, identified compounds, their respective molecular weights, and molecular structures. Stigmasterol, *ß*-sitosterol, and 3-O-acetyllupeol were the active compounds available in ETH and EHX in different percentages. The EHX fraction was found to have higher percentages of stigmasterol, *ß*-sitosterol, and 3-O-acetyllupeol with 4.41, 6.34, and 5.56%, respectively, while the percentages of the area in ETH extract were 2.49, 3.82, and 2.83%, respectively.

#### 3.4.2. Total Phenols

The total phenol content in the ethanol extract (ETH) and its fraction (EHX) was measured as *μ*g of gallic acid equivalent to mg of extract (GAE eq/mg). The total phenol content for ETH and EHX was 59.45 ± 1.46 and 64.59 ± 0.89 *μ*g GAE eq/mg extract, respectively (Table 7).

#### 3.4.3. Total Flavonoids

The total flavonoid content was measured as *μ*g of quercetin equivalent to mg of extract (QAE eq/mg extract). The result for ETH and EHX was 13.13 ± 0.05 and 32.10 ± 1.15 *μ*g QAE eq/mg extract, respectively (Table 7).

#### 3.4.4. DPPH Scavenging Activity

DPPH scavenging activity is presented as half-maximal inhibitory concentration (IC_50_) of ETH extract and its fraction EHX. The IC_50_ value for ETH and EHX was 62 ± 2.84 *μ*g/mL and 35.04 ± 0.21 *μ*g/mL, respectively. Ascorbic acid (Vit C) was used as a reference with IC_50_ of 3.83 ± 0.03 *μ*g/mL.

## 4. Discussion

Previously, reports have revealed the anticancer activity of *A. lappa* on various cell lines such as mouse hepatoma carcinoma cells (HepA) and sarcoma cells (S180), human breast cancer cells (MCF-7), gastric adenocarcinoma cells (BGC-823), mice spleen lymphocytes, and colorectal adenocarcinoma cells (Caco-2), [37, 38]. In this study, the aerial part of *A. lappa* was tested against two cancer cell lines (MCF-7 and HCT-116), and it is found to be more active against MCF-7.

According to Machado et al. [37], the ethanol extract from *A. lappa* and its ethyl acetate fraction inhibited the proliferation of Caco-2 better than the other extracts, and 3 subfractions out of 22 exhibited potent activity. Our results correspond well with the previous works on the anticancer activity of *A. lappa*. The ethanol extract (ETH) of *A. lappa* significantly inhibited the proliferation of MCF-7 compared to the other extracts. The IC_50_ value of ETH was 50.18 ± 3.66 *μ*g/mL. Consequently, the antiproliferative activity progressively improved in n-hexane fraction (EHX), which was obtained from the most active extract ETH. The efficacy of EHX was significantly better than other extracts and fractions against MCF-7 and EA.hy926. The IC_50_ values were 14.08 ± 3.64 and 27.25 ± 3.45 *μ*g/mL, respectively. Indeed, *A. lappa* contains many therapeutic constituents including lignans, terpenoids, and sterols, which have versatile biological activities targeting cancer and pathological angiogenesis [7, 39, 40]. The phytosterols (stigmasterol and *ß*-sitosterol) constituents have been proved in many studies as anticancer agents through various mechanisms of action, such as inhibiting cancer cell growth and inducing cancer cell apoptosis [40–42]. Therefore, it can be speculated that these compounds are a cause of the antiproliferative activity of *A. lappa,* and the different percentages of the area by the gas chromatogram may explain the lower IC_50_ of EHX compared to the ETH extract.

The Hoechst stain assay exhibited a clear morphological alteration, under a fluorescent microscope, in treated cells such as cell shrinkage, kidney shape, and apoptotic body compared to the untreated cells. This result was supported by an ultrastructural analysis from a transmission electron microscope. The microimages of treated cells illustrated the apoptotic changes compared to the control sample: condensed chromatin, floating nucleus content in the cytoplasm, apoptotic bodies, vacuoles with various sizes, and no nucleolus. This strongly suggests that cell death happened due to apoptosis induction in breast cancer cells [36, 43].

Moreover, the effect of EHX was tested on 10 cancer signal pathways related to the apoptosis and angiogenesis process. The data indicated that EHX significantly upregulated 3 pathways (p53, TGF *β*, and NFkB). p53 is a tumor suppressor gene that is critically involved in cell cycle regulation and apoptosis. [44]. The overexpression of p53 causes cell cycle arrest or induction of apoptotic cell death. p53 controls cell death through two apoptotic pathways: activation of death receptor DR-5 and Fas genes, which are related to the extrinsic pathway, and activation of Bak, Bax, and Bid proteins, which are related to the mitochondrial pathway [14]. This indicates that the proapoptotic activity of EHX against MCF-7 may be attributed to the activation of the p53 pathway.

TGF *β* is a major tumor suppressor, and the absence of this protein increases the risk of developing cancer. Pierce et al. [45] found that mice tend to be more resistant to mammary tumor formation when TGF *β* is hyperactivated, in addition to the tumor regression effect. Further researches suggested that the overexpression of TGF-*β* in breast cancer cells is able to suppress tumor development markedly [46, 47]. Our EHX fraction upregulated TGF *β* significantly in MCF-7. This might be a reason behind the anticancer efficacy of EHX towards MCF-7 cells.

NF-*κ*B acts as a guardian for malignant cells against apoptosis. Many studies have highlighted the important role of the NF-*κ*B pathway in cell growth, angiogenesis, and cell death suppression, and it is considered as a potential cancer drug target to curb malignancy [48]. Nevertheless, recent researches have challenged this idea because studies employing tumor models have shown that NF-*κ*B activation may also work against tumorigenesis. Interestingly, NF-*κ*B is directly connected to Fas expression, which is a member of the death receptor family. The canonical NF-*κ*B pathway was found to be a Fas transcription activator and it leads to induce apoptotic cell signals such as caspase 8 [49]. In another study, the nonsteroidal anti-inflammatory drug (aspirin) has been reported to upregulate this pathway through nuclear translocation of NF-*κ*B complexes to induce apoptotic cell death [50]. This result corresponds to the aspirin mechanism in apoptosis induction. *A. lappa* was reported to have anti-inflammatory properties, which are also evident in aspirin when exposed to cancer cells. This strongly suggests that the apoptotic cell death in MCF-7 may be initiated by the activation of the NF-*κ*B pathway [43, 51].

Further investigations were conducted on MCF-7 cells to understand the anticancer activity of EHX including colony formation, cell migration, and invasion assays. The metastatic cancer cells start with cell proliferation followed by migration. Cancer cells must be able to tolerate the hemodynamic stress and avoid the natural host immune response, and in order to reach distant organs, tumor cells have to adapt to the new microenvironment and start colonization along with a network of new blood vessels (angiogenesis) to supply them with vital oxygen and nutrients. Thus, failure in one of these steps can halt the metastatic tumor growth process. The colony formation assay was performed to determine whether the effect of EHX is cytotoxic or cytostatic [52]. The cell survival percentage of MCF-7 cells was zero at high concentration (7 *μ*g/mL), where EHX perturbs single cells to survive and to form colonies and thus confirms the cytotoxic effect. However, EHX at lower concentrations (1.75 and 0.875 *μ*g/mL) behaved as a cytostatic agent, where clonogenic cells started to revive upon the removal of EHX exposure. Cell migration was tested *in vitro* at two concentrations (15 and 30 *μ*g/mL). The lower concentration of EHX significantly inhibited MCF-7 to migrate to the other side of the wound during 6 h, but its inhibitory effect was markedly decreased after 12 h. Higher concentration had a stronger and significant inhibitory effect at all time points, and its wound remained open compared to the control sample, suggesting that EHX might disrupt the migration mechanism of breast cancer cells. Invasion of cancer cells into neighboring tissues and the vasculature plays an essential role in tumor metastasis. Cells tend to degrade the vascular subendothelial basement membrane to enter the bloodstream and to spread out to other body parts [53, 54]. Cell invasion assay was performed in the matrigel basement at concentrations of 7.5 and 15 *μ*g/mL. EHX inhibited the invasion of MCF-7 significantly at both concentrations. The results indicated that EHX might attenuate MCF-7 metastasis ability through the inhibitory effect in cell migration and invasion process. According to Awad et al. [6], phytosterols such as *ß*-sitosterol reduce the growth and metastasis of breast and pancreatic cancer cells in SCID mice, suggesting that the inhibitory effect of MCF-7 migration and invasion might be attributed to sterols constituents (stigmasterol and *ß*-sitosterol) of EHX.

A tumor can lie quiescent and clinically undetectable in the body for many years, but under certain circumstances, cancer cells can turn to the active phase and start to proliferate, metastasize, and induce angiogenesis. This switch may occur when the body's immune system is weakened or/and the stimulators such as VEGF (which regulate endothelial cell proliferation) and integrins (which control cell migration) outweigh the inhibitors [55]. The rat aorta ring assay was used to assess the antiangiogenic activity of EHX and the results revealed a potent inhibition with IC_50_ value 4.34 ± 1.64 *μ*g/mL. The process of angiogenesis is dependent on the presence of free radicals that help to activate the VEGF expression. VEGF production increases to obtain an adequate amount of oxygen in the tumor hypoxic region, leading to the activation of the angiogenesis process. This process is regulated by HIF-1*α* and HIF-1*β* which dimerize and translocate into the nucleus leading to the activation of VEGF expression [56]. The result of this study shows a decrease in VEGF protein expression levels in MCF-7 cells. The *ex vivo* studies using the rat aorta ring also show that EHX causes inhibition on neovascularization. So, the antiangiogenic activity of EHX may be acquired in consequence of the downregulation of VEGF expression. It is noteworthy that lupeol showed potent antiangiogenic properties in *in vitro* and *in vivo* studies and inhibited the expression of VEGF-A [8], and it could be behind the antiangiogenic activity of EHX. Furthermore, the effective role of oxidative stress in pathological angiogenesis related to tumors has been discovered through various connections and studies, elucidating that the oxidation mechanism plays a mediator role to release signals contributing in different angiogenic-related responses [57]. *A. lappa* is a plant rich with phenols and flavonoids which act as reducing agents by neutralizing the free radicals [58]. In this study, the total phenols, flavonoids, and antioxidant activity were measured, and the results have proved strong antioxidant properties. Therefore, the antiangiogenic activity may be attributed to the quenching ability of polyphenols present in this plant.

## 5. Conclusion

This study shows that *A. lappa* fraction (EHX) has an *in vitro* cytotoxic and proapoptotic effect towards MCF-7 breast cancer cells, and it can inhibit cell colonization, migration, and invasion. EHX upregulated 3 pathways (p53, TGF-*β*, and NF-k*β*) associated with an antitumor property. Furthermore, the antiangiogenic activity of *A. lappa* displayed a potent effect on microblood vessel outgrowth and it downregulated the VEGF-A expression. In conclusion, the present work reveals promising anticancer and antiangiogenic activity of *A. lappa*. Thus, further research is needed to determine the anticancer effect in animal models.

## Figures and Tables

**Table 1:** Percentage of cell viability by using MTT assay at a concentration 100 *μ*g/mL.

Extract	MCF-7	HCT-116	EA.hy926
Petroleum ether (PT)	29.25 ± 1.55^*∗*^	37.63 ± 3.27^*∗*^	59.75 ± 3.22^*∗*^
Ethanol (ETH)	22.95 ± 1.01^*∗*^	45.65 ± 4.82^*∗*^	
93.25 ± 1.05	35.53 ± 0.64^*∗*^		
Water (WT)	88.11 ± 2.2^*∗*^		89.97 ± 3.65^*∗*^
*n*-Hexane (EHX)	16.1 ± 1.81^*∗*^	—	22.97 ± 1.37^*∗*^
Ethyl acetate (EEA)	55.01 ± 2.37^*∗*^	—	60.77 ± 1.89^*∗*^
Butanol (EB)	70.63 ± 1.66^*∗*^	—	83.86 ± 2.14^*∗*^
Water (EW)	48.13 ± 2.98^*∗*^	—	56.68 ± 2.22^*∗*^
Negative control	100.01 ± 2.07	100.14 ± 0.25	100 ± 2.11

^*∗*^ *p* < 0.05 relative to control; values are mean ± SD.

**Table 2:** IC_50_ values (*μ*g/mL) by using MTT assay.

Extract	MCF-7	EA.hy926
ETH	50.18 ± 3.66	—
EHX	14.08 ± 3.64	27.25 ± 3.45
Tamoxifen	11.04 ± 1.31	—
Betulinic acid	—	4.27 ± 0.38

Values are mean ± SD.

**Table 3:** Rat aorta ring assay percentage of inhibition at 100 *μ*g/mL of extracts and the IC_50_ values (*μ*g/mL).

Extract	Inhibition percentage	IC_50_ value
Petroleum ether (PT)	45.88 ± 3.42^*∗*^	—
Ethanol (ETH)	59.75 ± 2.84^*∗*^	70.67 ± 3.06
Water (WT)	9.4 ± 1.5	—
*n*-Hexane (EHX)	100^*∗*^	4.34 ± 1.64
Ethyl acetate (EEA)	23.5 ± 3.61	—
Butanol (EB)	4.42 ± 1.33	—
Water (EW)	29.73 ± 4.08	—
Suramin (positive control)	—	4.62 ± 1.17

^*∗*^ *p* < 0.05 relative to control; values are mean ± SD.

**Table 4:** Interpretation of GC-MS analysis: identified compounds that are contained in both ETH and EHX.

RT/min	Compound	Molecular formula	Molecular weight	Area%
10.29	Bicyclo[3.1.1]heptane,-2,6,6-trimethyl-,(1.alpha.,2.*ß*.,5.alpha.)	C_10_H_18_	138.24	1.68–2.55
10.94	n-Hexadecanoic acid	C_16_H_32_O_2_	256.42	11.93–6.48
11.07	Ethyl palmitate	C_18_H_36_O_2_	284.47	2.15–1.01
11.67	Phytol	C_20_H_40_O	296.53	1.92–2.04
13.18	cis-(-)-2,4a,5,6,9a-Hexahydro-3,5,5,9-tetramethyl(1H)benzocycloheptene	C_15_H_24_	204.35	4.64–6.05
13.73	Dioctyl phthalate	C_24_H_38_O_4_	390.55	4.77–2.71
21.95	Stigmasterol	C_29_H_48_O	412.69	2.49–4.41
23.2	*ß*-Sitosterol	C_29_H_50_O	414.7	3.82–6.34
28.08	3-O-Acetyllupeol	C_32_H_52_O_2_	468.75	2.83–5.56

**Table 5:** Interpretation of GC-MS analysis: identified compounds that are contained in ETH only.

Peak no.	RT/min	Compound	Molecular formula	Molecular weight	Area%
1	5.03	Propane, 1,1,3-triethoxy-	C_9_H_20_O_3_	176.25	2.76
2	8.82	Diethyl phthalate	C_12_H_14_O_4_	222.23	1.96
3	9.99	Cyclononasiloxane, octadecamethyl-	C18H54O9Si9	667.38	1.13
5	10.32	3,7,11,15-Tetramethyl-2-hexadecen- 1-ol	C_20_H_40_O	296.53	0.67
6	10.54	Hexadecanoic acid, methyl ester	C_17_H_34_O_2_	270.45	0.41
9	11.40	7-Octadecenoic acid, methyl ester	C_19_H_36_O_2_	296.48	1.34
11	11.61	6-Octadecenoic acid	C_18_H_34_O_2_	282.46	18.55
12	11.71	Ethyl oleate	C_20_H_38_	310.51	13.66
13	11.80	Octadecanoic acid, ethyl ester	C_20_H_40_O_2_	312.53	1.42
15	12.48	Phenol, 4-(methylamino)-	C_7_H_9_NO	123.15	6.18
18	12.74	1,1,1,5,7,7,7-Heptamethyl-3,3-bis (trimethylsiloxy)tetrasiloxane	C_13_H_40_O_5_Si_6_	444.96	0.69
20	13.05	Naphthalene	C_10_H_8_	128.17	1.50
25	14.14	2,3-Dihydroxypropyl elaidate	C_21_H_40_O_4_	356.54	2.68

**Table 6:** Interpretation of GC-MS analysis: identified compounds that are contained in EHX only.

Peak no.	RT/min	Compound	Molecular formula	Molecular weight	Area%
5	11.25	Himachala-2,4-diene	C_15_H_24_	204.35	1.02
7	12.05	Parthenolide	C_15_H_20_O_3_	248.31	2.07
8	12.08	Arteannuin b	C_15_H_20_O_3_	248.31	3.61
9	12.35	Epiglobulol	C_15_H_26_O	222.36	4.20
10	12.54	4,4,5,8-Tetramethyl-chroman-2-one	C_13_H_16_O_2_	204.26	5.03
11	12.71	Pyridine, 2-butyl	C_9_H_13_ N	135.2	22.48
12	12.87	2,4,6-Trimethyl-1,3-phenylenediamine	(CH_3_)_3_C_6_H(NH_2_)_2_	150.22	0.91
13	13.15	1S,2S,5R-1,4,4-Trimethyltricyclo[6.3.1.0(2,5)]dodec-8(9)-ene	C_15_H_24_	204.35	5.77
15	13.32	Spathulenol	C_15_H_24_O	220.35	10.12
16	13.4	4-Camphenylbutan-2-one	C_14_H_22_O	206.32	4.79
18	15.65	Squalene	C_30_H_50_	410.71	1.32

**Table 7:** Antioxidant activity of ETH extract and its fraction EHX.

Extract	DPPH IC_50_ (*μ*g/mL)	Total phenols (*μ*g/mg)	Total flavonoids (*μ*g/mg)
ETH	62 ± 2.84	59.45 ± 1.46	13.13 ± 0.05
EHX	35.04 ± 0.21	64.59 ± 0.89	32.10 ± 1.15
Ascorbic acid	3.83 ± 0.03	—	—

Values are mean ± SD.

## Data Availability

The data used in this study to support our findings are available from the corresponding author upon request.

## References

[1] JianFeng C., PengYing Z., ChengWei X., TaoTao H., YunGui B., KaoShan C. (2012). Effect of aqueous extract of Arctium lappa L.(burdock) roots on the sexual behavior of male rats. *BMC Complementary and Alternative Medicine*.

[2] Cai Y., Luo Q., Sun M., Corke H. (2004). Antioxidant activity and phenolic compounds of 112 traditional Chinese medicinal plants associated with anticancer. *Life Sciences*.

[3] Lee J. H., Kim C., Lee J., Um J.-Y., Sethi G., Ahn K. S. (2019). Arctiin is a pharmacological inhibitor of STAT3 phosphorylation at tyrosine 705 residue and potentiates bortezomib-induced apoptotic and anti-angiogenic effects in human multiple myeloma cells. *Phytomedicine*.

[4] Khakdan F., Piri K., Talebi A. F. (2013). Antiproliferative activity of aqueous extract from Arictum lappa L. root in human erythroleukemia cell line (k562) and lymphocyte cell. *Life Sciences Journal*.

[5] Catalano E. (2013). Cytotoxic activity of a plant extract on cancer cells. *Italian Journal of Anatomy and Embryology*.

[6] Awad A. B., Fink C. S., Williams H., Kim U. (2001). In vitro and in vivo (SCID mice) effects of phytosterols on the growth and dissemination of human prostate cancer PC-3 cells. *European Journal of Cancer Prevention*.

[7] You Y.-J., Nam N.-H., Kim Y., Bae K.-H., Ahn B.-Z. (2003). Antiangiogenic activity of lupeol from Bombax ceiba. *Phytotherapy Research*.

[8] Avin B. V., Prabhu T., Ramesh C. K. (2014). New role of lupeol in reticence of angiogenesis, the cellular parameter of neoplastic progression in tumorigenesis models through altered gene expression. *Biochemical and Biophysical Research Communications*.

[9] National Institutes of Health; National Cancer Institute (2020). *Surveillance, Epidemiology, and End Results Program. Cancer stat facts: female breast cancer*.

[10] Waks A. G., Winer E. P. (2019). Breast cancer treatment. *JAMA*.

[11] Parise C. A., Caggiano V. (2014). Breast cancer survival defined by the ER/PR/HER2 subtypes and a surrogate classification according to tumor grade and immunohistochemical biomarkers. *Journal of Cancer Epidemiology*.

[12] Hengartner M. O. (2000). The biochemistry of apoptosis. *Nature*.

[13] Esposito F., Tornincasa M., Chieffi P., De Martino I., Pierantoni G. M., Fusco A. (2010). High-mobility group A1 proteins regulate p53-mediated transcription of Bcl-2 gene. *Cancer Research*.

[14] Yamasaki L., Frank D. A. (2003). Role of the RB tumor suppressor in cancer. *Signal Transduction in Cancer*.

[15] Wagner J., Kline C. L., El-Deiry W. (2017). Resistance to TRAIL pathway-targeted therapeutics in cancer. *TRAIL, Fas Ligand, TNF and TLR3 in Cancer*.

[16] Soleimani A., Khazaei M., Ferns G. A., Ryzhikov M., Avan A., Hassanian S. M. (2019). Role of TGF‐*β* signaling regulatory microRNAs in the pathogenesis of colorectal cancer. *Journal of Cellular Physiology*.

[17] Pei X., Li M., Zhan J. (2015). Enhanced IMP3 expression activates NF-кB pathway and promotes renal cell carcinoma progression. *PloS One*.

[18] Hopirtean C., Nagy V. (2018). Optimizing the use of anti VEGF targeted therapies in patients with metastatic colorectal cancer: review of literature. *Medicine and Pharmacy Reports*.

[19] Hassan L. E. A. (2014). Correlation of antiangiogenic, antioxidant and cytotoxic activities of some Sudanese medicinal plants with phenolic and flavonoid contents. *BMC Complementary and Alternative Medicine*.

[20] Lee M. S., Lee I. I., Kim Y., Kim Y. J., Heo H. J., Kim D.-O. (2014). Inhibitory effect of the ethyl acetate fraction from astringent persimmon on H2O2-induced oxidative stress in HepG2 cells. *Food Science and Biotechnology*.

[21] Baharetha H. M., Nassar Z. D., Aisha A. F. (2013). Proapoptotic and antimetastatic properties of supercritical CO2 extract of Nigella sativa Linn. against breast cancer cells. *Journal of Medicinal Food*.

[22] Hussaini S. Y., Haque R. A., Asekunowo P. O., Abdul Majid A. M. S., Taleb Agha M., Razali M. R. (2017). Synthesis, characterization and anti-proliferative activity of propylene linked bis-benzimidazolium salts and their respective dinuclear Silver(I)- N -heterocyclic carbene complexes. *Journal of Organometallic Chemistry*.

[23] Mu J., Liu T., Jiang L. (2016). The traditional Chinese medicine baicalein potently inhibits gastric cancer cells. *Journal of Cancer*.

[24] Frasca J. M., Parks V. R. (1965). A routine technique for double-staining ultrathin sections using uranyl and lead salts. *The Journal of Cell Biology*.

[25] Franken N. A. P., Rodermond H. M., Stap J., Haveman J., van Bree C. (2006). Clonogenic assay of cells in vitro. *Nature Protocols*.

[26] Fatima T., Haque R. A., Iqbal M. A. (2017). Tetra N -heterocyclic carbene dinuclear silver(I) complexes as potential anticancer agents: synthesis and in vitro anticancer studies. *Journal of Organometallic Chemistry*.

[27] Hamidi A., Song J., Thakur N. (2017). TGF-*β* promotes PI3K-AKT signaling and prostate cancer cell migration through the TRAF6-mediated ubiquitylation of p85*α*. *Science Signaling*.

[28] Tabana Y. M., Al-Suede F. S. R., Khadeer M. B. (2016). Cat’s whiskers (Orthosiphon stamineus) tea modulates arthritis pathogenesis via the angiogenesis and inflammatory cascade. *BMC Complementary and Alternative Medicine*.

[29] Bocci G., Danesi R., Benelli U. (1999). Inhibitory effect of suramin in rat models of angiogenesis in vitro and in vivo. *Cancer Chemotherapy and Pharmacology*.

[30] Kitson F. G., Larsen B. S., McEwen C. N. (1996). *Gas Chromatography and Mass Spectrometry: A Practical Guide*.

[31] Lizcano L. J., Bakkali F., Begoña Ruiz-Larrea M., Ignacio Ruiz-Sanz J. (2010). Antioxidant activity and polyphenol content of aqueous extracts from Colombian Amazonian plants with medicinal use. *Food Chemistry*.

[32] Baharetha H., Nassar Z. D. (2016). Use of nigella sativa linn. supercritical carbon dioxide extract for targeting the angiogenesis cascade. *Medicinal & Aromatic Plants*.

[33] Tabana Y. M., Dahham S. S., Ahmed Hassan L. (2015). In Vitro anti-metastatic and antioxidant activity of nicotiana glauca fraction against breast cancer cells. *Advances in Biological Regulation*.

[34] Dahham S. S. (2015). Antioxidant activities and anticancer screening of extracts from banana fruit (Musa sapientum). *Academic Journal of Cancer Research*.

[35] Cheah Y. H., Azimahtol H. L. P., Abdullah N. R. (2006). Xanthorrhizol exhibits antiproliferative activity on MCF-7 breast cancer cells via apoptosis induction. *Anticancer Research*.

[36] Mao X.-M., Zhou P., Li S.-Y. (2019). Diosgenin suppresses cholangiocarcinoma cells via inducing cell cycle arrest and mitochondria-mediated apoptosis. *OncoTargets and Therapy*.

[37] Machado F. B., Yamamoto R. E., Zanoli K. (2012). Evaluation of the antiproliferative activity of the leaves from Arctium lappa by a bioassay-guided fractionation. *Molecules*.

[38] Foldeak S., Dombradi G. (1964). Tumor-growth inhibiting substances of plant origin. I. Isolation of the active principle of Arctium lappa. *Acta Physico-Chimica*.

[39] Liu C., Srivastava K. D., Yang N. (2016). Arctigenin isolated from Arctium lappa L. Inhibits IgE production. *Journal of Allergy and Clinical Immunology*.

[40] Ali H., Dixit S., Alqahtani S., Ali D., Alkahtani S., Alarifi S. (2014). Isolation and evaluation of anticancer efficacy of stigmasterol in a mouse model of DMBA-induced skin carcinoma. *Drug Design, Development and Therapy*.

[41] Woyengo T. A., Ramprasath V. R., Jones P. J. H. (2009). Anticancer effects of phytosterols. *European Journal of Clinical Nutrition*.

[42] Park C., Moon D.-O., Rhu C.-H. (2007). *β*-Sitosterol induces anti-proliferation and apoptosis in human leukemic U937 cells through activation of caspase-3 and induction of bax/bcl-2 ratio. *Biological & Pharmaceutical Bulletin*.

[43] Toné S., Sugimoto K., Tanda K. (2007). Three distinct stages of apoptotic nuclear condensation revealed by time-lapse imaging, biochemical and electron microscopy analysis of cell-free apoptosis. *Experimental Cell Research*.

[44] Labuschagne C. F., Zani F., Vousden K. H. (2018). Control of metabolism by p53 - cancer and beyond. *Biochimica et Biophysica Acta (BBA)—Reviews on Cancer*.

[45] Pierce D. F., Gorska A. E., Chytil A. (1995). Mammary tumor suppression by transforming growth factor beta 1 transgene expression. *Proceedings of the National Academy of Sciences*.

[46] Katsuno Y., Lamouille S., Derynck R. (2013). TGF-*β* signaling and epithelial-mesenchymal transition in cancer progression. *Current Opinion in Oncology*.

[47] Roberts A. B., Wakefield L. M. (2003). The two faces of transforming growth factor in carcinogenesis. *Proceedings of the National Academy of Sciences*.

[48] Barlett M., Gilmore T. (1999). Control of apoptosis by Rel/NF-*κ*B transcription factors. *Oncogene*.

[49] Liu F., Bardhan K., Yang D. (2012). NF-*κ*B directly regulates Fas transcription to modulate fas-mediated apoptosis and tumor suppression. *Journal of Biological Chemistry*.

[50] Stark L. A., Din F. V. N., Zwacka R. M., Dunlop M. G. (2001). Aspirin-induced activation of the NF-*κ*B signaling pathway: a novel mechanism for aspirin-mediated apoptosis in colon cancer cells. *The FASEB Journal*.

[51] Kerr J. F. R. (2002). History of the events leading to the formulation of the apoptosis concept. *Toxicology*.

[52] Plumb J. A. (2004). *Cancer Cell Culture: Methods and Protocols*.

[53] Yamaguchi H., Wyckoff J., Condeelis J. (2005). Cell migration in tumors. *Current Opinion in Cell Biology*.

[54] Mader C. C., Oser M., Magalhaes M. A. O. (2011). An EGFR-src-arg-cortactin pathway mediates functional maturation of invadopodia and breast cancer cell invasion. *Cancer Research*.

[55] Folkman J. (2002). Role of angiogenesis in tumor growth and metastasis. *Seminars in Oncology*.

[56] Ahluwalia A., Tarnawski A. S. (2012). Critical role of hypoxia sensor - HIF-1α in VEGF gene activation. Implications for angiogenesis and tissue injury healing. *Current Medicinal Chemistry*.

[57] Kovacs K., Marra K. V., Yu G. (2015). Angiogenic and inflammatory vitreous biomarkers associated with increasing levels of retinal ischemia. *Investigative Opthalmology & Visual Science*.

[58] Yehya A. H. (2017). Broad spectrum targeting of tumor vasculature by medicinal plants: an updated review. *Journal of Herbal Medicine*.

